# Perceptions and responses to cognitive decline in people with diabetes: A systematic review of qualitative studies

**DOI:** 10.3389/fpubh.2023.1076030

**Published:** 2023-02-17

**Authors:** Meijuan Wang, Xiangyun Guan, Jingzheng Yan, Nyagwaswa Michael, Xueyan Liu, Ran Tan, Xiaoyan Lv, Fei Yan, Yingjuan Cao

**Affiliations:** ^1^School of Nursing and Rehabilitation, Shandong University, Jinan, China; ^2^Department of International Medical Department, Qilu Hospital of Shandong University, Jinan, China; ^3^Department of Breast Surgery, Qilu Hospital of Shandong University, Jinan, China; ^4^Department of Endocrinology, Qilu Hospital of Shandong University, Jinan, China; ^5^Department of Nursing, Qilu Hospital of Shandong University, Jinan, China; ^6^Nursing Theory and Practice Innovation Research Center, Shandong University, Jinan, China

**Keywords:** diabetes mellitus, cognitive decline, perception, qualitative research, systematic review

## Abstract

**Objective:**

We aimed at summarizing the perceptions and responses to cognitive decline, assessing the disease management, identifying deficiencies and proposing new strategies for improvement in people with diabetes (PWDs).

**Methods:**

A comprehensive search was performed in the following nine databases: PubMed, EMBASE, Web of Science, The Cochrane Library, PsycINFO, CINAHL, WanFang, CNKI, and VIP. The Joanna Briggs Institute (JBI) Critical Appraisal Tool for qualitative research was utilized to evaluate the quality of included studies. Descriptive texts and quotations relating to patient experience were extracted from the included studies and thematically analyzed.

**Results:**

Eight qualitative studies met the inclusion criteria and 2 overarching themes were identified: (1) self-perception of cognitive decline referred to perceived cognitive symptoms, lack of knowledge and, impaired self-management and coping in multiple methods; (2) reported benefits of cognitive interventions referred to how cognitive interventions improved disease management, attitudes and needs of PWDs.

**Conclusion:**

PWDs described misconceptions about their cognitive decline and suffered from them during disease management. This study provides a patient-specific reference for cognitive screening and intervention in PWDs, supporting disease management with cognitive decline in clinical practice.

## 1. Introduction

Diabetes mellitus is a chronic metabolic disease that damages human health (1). Owing to an aging population and the growing number of people with obesity, diabetes has affected ~537 million people worldwide with an increasing prevalence (2). The comorbidities accompanied with diabetes also deteriorate people's quality of life, especially cognitive impairment which is associated with increasing risk of mortality (3). Studies have shown that cognitive decline in people with diabetes (PWDs) progresses twice as fast as normal aging, and more likely develops into Alzheimer's disease and dementia (4–6).

PWDs have to hold complex and perpetual self-management to maintain their health and independent lifestyle, all of which may be disorganized by cognitive decline (7, 8). PWDs with worse self-management and other complications such as hypoglycemia and depression are more prone to cognitive impairment (9). A recent population-based cohort study suggested that poorly controlled diabetes was associated with double the risk of cognitive impairment and triple the risk of cognitive impairment progressing to dementia (10). PWDs will be caught in a vicious circle and unable to coexist with the disease along with decreasing quality of life, shorter life expectancy and higher mortality due to cognitive decline (11, 12). Therefore, it is critical for PWDs to timely and effectively improve and strength their disease management ability weakened due to cognitive decline.

Although cognitive decline can have deleterious effects, it is not irreparable. Studies have shown that ~10–40% of people with mild cognitive impairment (MCI) may return to normal cognitive performance within ~4–5 years (13). Cognitive interventions may also have a positive effect on cognitive decline in PWDs (14). However, there are various types of cognitive impairment, and different people have great heterogeneity in the symptoms they perceive and the ways they deal with cognitive impairment. A number of studies have explored the impact of cognitive decline on specific components of self-management in PWDs. Evidence has shown that global cognitive decline is mainly associated with poor medication management in PWDs (15), such as lower insulin self-injection knowledge (16), less responsibility for self-medication (17), improper filling (18) and being less likely to take oral medications on time (19). PWDs are also less likely to engage in glucose self-monitoring and use health care clinics properly (17, 19). Furthermore, a significant association was found between global cognitive decline and diet adherence (20). Nevertheless, specific symptoms and performances of disease management among PWDs with cognitive decline have not been systematically evaluated and synthesized.

Neuropsychological tests are now commonly used to assess PWDs' cognitive function. Common instruments are based on theoretical knowledge, statistical methods and diagnostic criteria, without a systematic qualitative research, which probably leads to omissions when mild cognitive changes are assessed (21, 22). Qualitative methods have the strength of addressing highly nuanced and contextualized aspects of a subjective experience (23). There is already a substantial literature dealing with the qualitative exploration of the lived experience of people with dementia (24–27). However, the qualitative description of the experiences underlying cognitive complaints has only recently been pursued with PWDs.

This study aimed at summarizing the perceptions and responses to cognitive decline in PWDs, assessing the disease management, identifying deficiencies and proposing new strategies for improvement.

## 2. Methods

This study adhered to the Preferred Reporting Items for Systematic review and Meta-Analysis guideline (PRISMA) (28). The study protocol was registered in PROSPERO with the registration number CRD42022301334.

### 2.1. Data source and search strategy

Two independent reviewers performed a comprehensive search in the following nine databases: PubMed, EMBASE, Web of Science, The Cochrane Library, PsycINFO, CINAHL, WanFang, CNKI, and VIP. The search period ended in January 2022. Search strategies for all databases are listed in Supplementary Table S1.

### 2.2. Eligibility criteria

We included qualitative studies conducted in individual with type 1 diabetes or type 2 diabetes published in a peer-reviewed journal in either English or Chinese language. Studies including people without diabetes were considered only if they specifically reported results for PWDs. The included studies should examine perceptions and/or experiences of cognitive decline, thoughts, attitudes, feelings and views. Studies were excluded if they did not meet the above criteria.

### 2.3. Data selection and extraction

Two reviewers independently screened all papers according to the eligibility criteria (defined earlier), extracted and cross-checked the data. The following information was extracted: the surname of the first author and publication year; location; sample; PWDs' age; method/theory; data collection methods; research objective. Themes from the study's result section and participants' direct quotations were extracted as findings. For studies without direct quotes, the researchers extracted appropriate text after repeatedly reading the narrative. Extracted data were then imported into MS Excel for further coding and integration. Discrepancies between reviewers were resolved through discussion or by referring to a third reviewer.

### 2.4. Quality assessment

The methodological quality of the included studies was independently assessed for quality by two reviewers using the criteria based on the JBI Critical Appraisal Tool for qualitative research. Each checklist item was graded as “Yes,” “No,” and “Unclear.” The two reviewers shared the results of the checklist and arrived at a consensus.

### 2.5. Data synthesis and analysis

Thematic synthesis was used to analyze the qualitative data from the included papers (29). Two reviewers coded text fragments for similarity. All extracted results were read repeatedly to extract concepts for coding. Individual codes were then combined into groups and summarized by descriptive themes. Subthemes were used to further refine and categorize descriptive themes. Finally, distinct analytical themes were defined. The contents were organized into a structured hierarchy reflecting the content of the included studies. Distinct analytical themes were defined. The synthesized results reinforced the of current knowledge and generated new insights.

## 3. Results

### 3.1. Study selection

**Figure 1:**
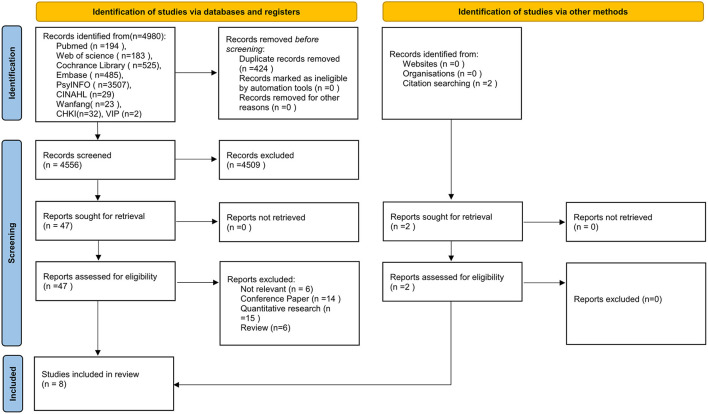
Flowchart of the study.

The initial search produced 4,556 articles after excluding duplicates, which were further reduced by 4,509 after excluding articles based on reviewing the titles and abstracts. Full texts of the remaining 49 articles were retrieved and a further 41 articles were excluded after review. Figure 1 shows the document selection process. Two of the papers were selected from the reference list of the included studies. Overall, eight qualitative studies were selected for inclusion.

### 3.2. Study characteristics

Inclusive study characteristics are shown in Table 1. Studies were conducted in the United Kingdom (*n* = 2), United States (*n* = 3), China (*n* = 1), Germany (*n* = 1), and New Zealand (*n* = 1). All included studies reported their sample size, which varied from 7 to 30 participants. All eight papers had participants with diabetes making a total sample size of 127. The JBI total score for each study is provided in Table 2. The quality of included studies varied considerably, with scores ranging from 60 to 90%.

**Table 1:** Characteristics of included studies.

**References**	**Location**	**Sample**	**Age (years)**	**Methodology/** **theory orientation**	**Method of data collection**	**Research questions or objectives**
Hasseler et al. (30)	Germany	7 T2DM patients	55–76	Symbolic interactionism	Problem-centered interview	To identify diabetes-related coping strategies and problems of adjustment to the disease from the perspective of PWDs.
Wilson (31)	UK	25 T1DM or T2DM patients	72–84	None stated	Telephone interview	To explore the views of older PWDs about the care they received from healthcare professionals.
Speight et al. (32)	UK	17 T1DM patients	35–57	Adapted grounded theory	Semi-structured interview	To explore individual experiences of severe hypoglycaemia occurring in daily life, and understand barriers to the prevention of hypoglycaemia.
Cuevas et al. (33)	USA	10 T2DM patients	44–70	None stated	Narrative interview	To explore the perceptions of people with T2DM regarding cognitive changes they experienced and examine informants' recommendations for modifications of existing cognitive rehabilitation interventions.
Cuevas et al. (34)	USA	19 T2DM patients	40–70	None stated	Focus group	To describe and focus specifically on the perceptions of people with T2DM in a cognitive rehabilitation intervention.
Hu and Zhang (35)	China	9 T2DM patients	41–57	Phenomenology	Semi-structured interview	To explore the cognition and feeling of MCI in PWDs, evaluate their performance and demand for cognitive rehabilitation intervention.
Chepulis et al. (36)	New Zealand	10 T2DM patients	26–75	None stated	Semi-structured interview	To provide an up-to-date assessment of challenges to diabetes care and glycemic control, particularly in patients with T2DM who have severe glycemic control.
Cuevas et al. (37)	USA	30 T2DM patients	Mean age = 66	None stated	One-on-one interview	To explore the meaning of cognitive health from the perspectives of Latinx adults with T2DM.

NB: T2DM, Type 2 diabetes mellitus; T1DM, Type 1 diabetes mellitus.

**Table 2:** Quality assessments.

**References**	**Criteria**										**Score (%)**
	**Q1**	**Q2**	**Q3**	**Q4**	**Q5**	**Q6**	**Q7**	**Q8**	**Q9**	**Q10**	
Hasseler et al. (30)	U	Y	Y	Y	Y	Y	N	N	N	Y	60%
Wilson (31)	U	Y	Y	Y	Y	N	N	Y	Y	Y	70%
Speight et al. (32)	U	Y	Y	Y	Y	Y	Y	Y	Y	Y	90%
Cuevas et al. (33)	Y	Y	Y	Y	Y	U	Y	Y	Y	Y	90%
Cuevas et al. (34)	U	Y	Y	Y	Y	U	Y	Y	Y	Y	80%
Hu and Zhang (35)	Y	Y	Y	Y	Y	N	U	Y	Y	Y	80%
Chepulis et al. (36)	U	Y	Y	Y	Y	Y	U	Y	Y	Y	80%
Cuevas et al. (37)	Y	Y	Y	Y	Y	Y	U	Y	Y	Y	90%

Y, Yes; N, No; U, Unclear; Q1, Congruity between the stated philosophical perspective and the research methodology; Q2, Congruity between the research methodology and the research question or objectives; Q3, Congruity between the research methodology and the methods used to collect data; Q4, Congruity between the research methodology and the representation and analysis of data; Q5, There is congruence between the research methodology and the interpretation of results; Q6, Locating the researcher culturally or theoretically; Q7, Influence of the researcher on the research, and vice-versa, is addressed; Q8, Representation of participants and their voices; Q9, Ethical approval by an appropriate body; Q10, Relationship of conclusions to analysis, or interpretation of the data.

### 3.3. Data synthesis

Two overarching themes were identified: self-perception of cognitive decline, and reported benefits of cognitive interventions. Supplementary Table S2 provides an overview of the two overarching themes and their sub-themes, with illustrative quotes from participants and a list of the codes.

#### 3.3.1. Self-perception of cognitive decline

##### 3.3.1.1. Underestimatione of cognitive decline associated with diabetes

Two studies reported participants' dissociative cognitive decline with diabetes and their unawareness of the link between diabetes and cognitive decline (33, 35). Unsurprisingly, cognitive problems were attributed to aging and considered to be a normal part of the aging process, albeit some participants were not elderly (33–35, 37). Persons appeared to treat the corresponding symptoms as common and ordinary and not as a problem with their cognitive health (37). In three studies, health care providers did not provide information about the association between diabetes and cognitive decline, which participants rarely acquired through other means (34, 35, 37). Only a few participants knew a little about the dangers of hypoglycaemia on cognitive function (33, 35).

##### 3.3.1.2. Suffering from cognitive symptoms

The most common symptom was deterioration in memory capacity. PWDs found themselves had significantly reduced memory capacity, especially short-term memory (32–35). Inadequate attention was another common symptom, with participants reporting the inability to concentrate and lack of interest (32). PWDs reported difficulties in generating thoughts and responses and/or maintaining psychomotor skills (31, 32, 37). In a single study, some PWDs who were aware of their cognitive dysfunction developed a sense of shame, remaining silent for fear of stigma (32).

##### 3.3.1.3. Impaired diabetes self-management

Cognitive decline causes many difficulties for PWDs in the disease management and their daily activities. This extends to patients' ability to use and implement new knowledge in their everyday life (30). Medication non-adherence was a common finding as participants forgot to take their medication, resulting in unstable blood glucose levels and hospitalization (31, 33, 35, 36). A planned diets also became difficult to implement as plans are time-consuming and easy to forget (32, 33, 35). Participants often forgot the sequence of care routines leading to a loss of confidence in self-management of the disorder (31, 37). In addition, cognitive decline affected the their ability to care for family, work, and maintain social relationships, leading to problems with social functioning and the patient's quality of life (37).

##### 3.3.1.4. Coping in multiple methods

Although cognitive decline disabling, many PWDs devised useful ways to actively cope with the problem. Some PWDs educated themselves through books or online to compensate for their lack of knowledge on the disease and the knowledge from health care providers (33, 37). Many PWDs implemented compensatory strategies such as list-making or other mnemonic devices to mitigate deficits in attention/orientation (33, 34, 37). This ensured work or life balance (37). Many other methods were used to promote cognitive health such as having a hobby, doing physical activity, playing video games, dieting and having social interactions (33, 37). Participants often acknowledged the cognitive benefits of these approaches without verifying whether these methods really work.

#### 3.3.2. Reported benefits from cognitive interventions

##### 3.3.2.1. Benefiting from cognitive interventions

PWDs received cognitive interventions in two studies, including educational sessions teaching compensatory cognitive strategies and online brain training programs (33, 34). They benefited from these interventions, supporting an improved diabetes management. Some participants learned the content of the intervention and applied the cognitive strategies to their long-term practice (33). After taking educational sessions, PWDs used to interact better with their health care providers, for example asking to assess a potential vitamin B12 deficiency associated with metformin use or requesting to perform additional tests for the measurement of cognitive deficits (34). Through practice of cognitive strategies in the class, PWDs were also facilitated to think about and use cognitive strategies in the process of disease self-management thus helping them to manage their progress as well as consider the basis for many other methods (34). Some PWDs realized that the effects of the intervention were both short-term and long-term. The short-term effect resulted from learning to think better and using cognitive strategies, while the long-term effect was related to better diabetes management such as improved blood glucose, cholesterol, blood pressure, and cognitive function (34). Most participants had a sense of achievement and felt that the intervention had helped to improve their “mental capacity and flexibility in planning cognitive strategies” (34).

##### 3.3.2.2. Attitude to cognitive interventions

Most participants had a positive attitude and a strong interest in cognitive interventions. They anticipated learning a myriad of better ways of cognitive brain function through the intervention, believing it would improve cognition (33). In addition, most participants appeared to appreciate the clarification of cognitive content, as information overload proved overwhelming as it introduced uncertainty and fears about being misled. The ability of health care providers to clarify this information made them feel reassured and helpful (34). Participants were not just interested in cognitive interventions but appeared willing and ready to participate (33). PWDs' attitudes were also influenced by the content and form of the intervention (33). Mainly, PWDs had intrinsic and extrinsic motivations to participate in the intervention. Encouragement and support from health care professionals also were important drivers (34).

However, significant barriers also existed, such as the time factor. People's attendance was adversely affected when course or group meeting times clashed with participants' working hours or appointments (33, 35). Additionally, some people had difficulty planning or implementing their plans, which prevented them from attending sessions on time (34). Others found it difficult to change habits. Consequently, this made the intervention ineffective and caused participants to lose faith in the intervention, and finally, drop out of the sessions (34).

##### 3.3.2.3. Preference for cognitive interventions

PWDs were expected to learn better cognitive strategies to improve cognitive functioning (34, 37). A wide range of content was recommended as specific areas of interest, which included understanding how diabetes affects cognitive function, cognitive decline coping skills during diabetes self-management, discussing the association between diabetes-related stress and cognitive function, and learning how to integrate a “brain-healthy” lifestyle, especially as it relates to diet, in routine activities (33). Participants appeared keen to understand the link between diabetes and cognitive decline and be informed at the time of diagnosis for early preparation for the onset of cognitive problems (33). PWDs with cognitive problems needed information about treatment options such as diet and medication (35). The studies also recommended focusing on teaching cognitive strategies that can improve quality of life (33). Patients' inattentiveness as a result of cognitive dysfunction creates a sense of helplessness over their illness and triggers anxiety, therefore exercises are required to improve these problems as a matter of priority such as meditation and deep breathing (34). Some people preferred a group format for interventions, as they wanted to “learn cognitive strategies from each other and share ideas” and acquire new knowledge (33).

## 4. Discussion

This is the first study that systematically reviewed the performances of disease management in PWDs during their cognitive decline. The findings showed that PWDs often experienced cognitive symptoms without recognizing pre-existing cognitive decline. In terms of self-response, they tried various methods to deal with cognitive decline with no certainty that these approaches could be effective. The cognitive interventions supported PWDs with increasing knowledge and practical strategies to better manage their disease.

In our findings, PWDs often perceived their own cognitive problems they were experiencing as normal part of aging, although some people did not fit this profile. Consistently with our findings, a review of the qualitative literature found that normal aging was the most common cause attribute to the self-perceived cognitive changes (21). Age, vascular and metabolic risk factors are related to mild cognitive impairment and dementia (38). As a most common metabolic risk factor, diabetes may aggravate cognitive decline with age, which is obviously ignored by PWDs. Lack of knowledge prevents them from recognizing cognitive decline in time and reduces the efficacy of self-management, including medication compliance, a proper diet and the application of new knowledge (39). Therefore, providing a timely education to PWDs may support them in preventing and managing the development of cognitive impairment.

Although PWDs reported many methods used to cope with cognitive decline, these methods appear to be applicable to various scenarios of daily life and not be diabetes-specific. Our results cannot reveal how PWDs adapt diabetes self-management strategies to cope with cognitive decline, which requires more in-depth research to explore. However, it may also suggest that PWDs cannot cope with the disruptions in diabetes management caused by cognitive decline on their own and they need external help and support.

Furthermore, based on our findings, PWDs with cognitive impairment did not receive adequate medical, educational, or emotional support from their health care providers. Although they adapted a variety of methods to help themselves cope with difficulties caused by cognitive impairment, support from health care providers means a lot to them. There are several possible reasons for this. First, a lack of awareness and comprehension of the co-occurrence of these disorders among health and social care providers might result in their inability to recognize and promptly treat cognitive impairment (40). Health and social care providers generally lack the awareness of the bidirectional relationship between diabetes management and cognitive impairment, resulting in an increased risk of diagnostic and treatment deficits (40, 41). Although the benefits of routine cognitive screening in PWDs have not been determined (42), health care providers should be alerted to memory complaints developed in PWDs or their caregivers. Thus, health care workers need to improve their understanding of the relationship between diabetes and cognitive impairment to reduce the impact of the disease and ameliorate clinical outcomes.

Secondly, although several guidelines have provided some relevant management recommendations, there is a lack of a comprehensive guidance for the clinical management of patients with diabetes and cognitive impairment. The American Diabetes Association (43), a UK Multidisciplinary National Expert Working Group (40), the American Association of Diabetes Educators (44) and the Chinese Medical Association (45) provide optimal practice guidance for healthcare professionals caring for patients with diabetes combined with cognitive impairment or dementia. Global guidelines from the International Diabetes Federation on managing older patients with type 2 diabetes provide the earliest suggestions for looking after patients with different functional deprivations, including frailty and dementia. While the guidelines have been well-received, their recommendations are not based on evidence of effectiveness in clinical practice (46). These guidelines need to be updated and improved as more contemporary evidence-based results become available.

Currently, there is no specific treatment plan for PWDs with mild cognitive impairment or dementia. The novel SGLT2 inhibitors have the potential to prevent and improve the cognitive decline associated with type 2 diabetes. The mechanisms underlying the development of cognitive impairment in PWDs have not been fully elucidated, but the available evidence suggests a possible combination of vascular damage, chronic inflammation and neurodegenerative pathology (11). Animal experiments showed that SGLT2 inhibitors have neuroprotective, anti-inflammatory, oxidative stress-reducing and anti-atherosclerotic effects (47). Carmen et al. found that empagliflozin reduced vascular damage and cognitive impairment in a mixed murine model of Alzheimer's disease and type 2 diabetes (48). However, we have little information on how SGLT2 inhibitors affect cognitive decline in clinical diabetes (49). Serena et al. found that a SGLT2 inhibitor was positively associated with better cognitive scores in a cohort of patients with diabetes (50). A prospective study showed significant beneficial effects of empagliflozin on cognitive and physical decline in frail older adults with diabetes and heart failure with preserved ejection fraction (51). New studies are needed to substantiate the benefits of SGLT2 inhibitors on cognitive impairment in people with type 2 diabetes.

Cognitive training was beneficial in PWDs. Cognitive training is a common non-pharmacological intervention to treat people with cognitive impairment (45). An eight-week, nurse-led study of a cognitive training intervention conducted in people with type 2 diabetes found that 58% of participants stated the intervention helped their diabetes self-management, and 74% expressed the desire to continue using the learned cognitive strategies (52). Another online cognitive intervention study found that individuals with diabetes improved scores on self-management, cognition and self-efficacy, with an increased adherence to a proper diet and medications (53). These findings are consistent with our results. We showed that PWDs were particularly interested in cognitive training, as well as lifestyle interventions aimed at improving cognitive function. Cognitive training allows PWDs to gain a sense of accomplishment, learn new skills and reduce anxiety. In previous studies, cognitive training improved cognitive skills or daily activities in the average population with mild cognitive impairment or mild to moderate dementia (54, 55). However, some studies have shown different results. Wong et al. found that the combination of patient empowerment and cognitive training did not improve glycemic control or self-care activities in older PWDs with memory complaints (56). A systematic review found moderate strength of evidence that cognitive training may improve performance in trained cognitive domains (57). The reasons for these opposite findings may be due to different study participants and methodological discrepancies, including incompatible treatments and dissimilar treatment durations. Large-scale and high-quality studies are needed in the future to demonstrate the types of cognitive interventions that can be successfully utilized in clinical practice.

## 5. Strengths and limitations

To our knowledge, this is the first qualitative systematic review that specifically addresses cognitive problems in PWDs by identifying how they perceive and experience their cognitive problems and describing the impact of cognitive problems on their daily lives. The explicit and comprehensive search strategy reported quality appraisal of the included studies and data synthesis process. Nevertheless, the study had several limitations. The first is the small sample size of the included articles. However, the included articles were heterogeneous in design so they were representative of diverse patient populations although they cannot be generalized. Second, we included three studies published by Cuevas et al., suggesting a potential bias. However, we conducted a comprehensive literature search with a rigorous screening, ensuring the reliability of our results. The thematic analysis was an interpretative process and the outcomes were validated by the co-authors, thus there was the potential for other interpretations. Finally, the exclusion of languages other than English or Chinese meant relevant studies published in other languages may have been overlooked.

## 6. Conclusions

This study showed that cognitive problems often occur among PWDs, seriously affecting their self-management and daily life activities. This phenomenon has received little attention from healthcare professionals, with limited patient education or treatment interventions. Given the deleterious effects of cognitive impairment on PWDs, healthcare providers should focus more on cognitive performance, facilitating and supporting treatments and interventions for cognitive impairment. Health education in PWDs might help them self-monitor and identify cognitive impairment. New studies will explore effective cognitive interventions in large-scale trials.

## Author contributions

YC, MW, and XG contributed to the study design, data acquisition, and analysis and manuscript revision. MW, XG, and JY contributed to data acquisition, interpretation of data, and manuscript revision. NM, RT, and XLi contributed to quality assessments, manuscript drafting, and revision. XLv and FY contributed to manuscript drafting and revision. YC is the guarantor of this work. All authors significantly contributed to the manuscript and approved the final version for publication.
