# Supplementary material for: Perceptions and responses to cognitive decline in people with diabetes: A systematic review of qualitative studies
Source: Front Public Health. 2023 Feb 17;11:1076030. doi: 10.3389/fpubh.2023.1076030 (PMC9981946; doi:10.3389/fpubh.2023.1076030)
Supplement: Supplementary file 1 [file Table_1.DOCX]

Supplementary Material

# Supplementary table 1. Search strategies

| **Source** | **Search strategy** |
| --- | --- |
| **PubMed** | #1 "Cognitive Dysfunction" [MeSH Terms] OR "cognitive dysfunction" [Title/Abstract] OR "mild cognitive dysfunction" [Title/Abstract] OR "cognitive impairment" [Title/Abstract] OR "mild cognitive impairment" [Title/Abstract] OR "MCI" [Title/Abstract] OR "cognitive disorder" [Title/Abstract] OR "Cognition Disorders" [Title/Abstract] OR "mild cognitive disorder" [Title/Abstract] OR "cognitive decline" [Title/Abstract] OR "mild cognitive decline" [Title/Abstract] OR "MCD" [Title/Abstract] OR "cognitive defect" [Title/Abstract] OR "Neurocognitive Disorders" [MeSH Terms] OR "neurocognitive disorder" [Title/Abstract] OR "mental deterioration" [Title/Abstract] OR "Mental Disorders" [Title/Abstract] OR "neurocognitive disorders" [Title/Abstract]  #2 "Diabetes Mellitus" [MeSH Terms] OR "Diabetes Mellitus, Type 1" [MeSH Terms] OR "Diabetes Mellitus, Type 2" [MeSH Terms] OR "diabetes mellitus" [Title/Abstract] OR "DM" [Title/Abstract] OR "diabet*" [Title/Abstract] OR "Diabetes Mellitus, Type 1" [Title/Abstract] OR "Diabetes Mellitus,Insulin-Dependent" [Title/Abstract] OR "IDDM" [Title/Abstract] OR "type 1 diabetes" [Title/Abstract] OR "T1DM" [Title/Abstract] OR "Diabetes Mellitus, Type 2" [Title/Abstract] OR "Diabetes Mellitus,Noninsulin-Dependent" [Title/Abstract] OR "type 2 diabetes" [Title/Abstract] OR "T2DM" [Title/Abstract] OR "NIDDM" [Title/Abstract]  #3 "experien*" [Title/Abstract] OR "Perception" [MeSH Terms] OR "perception*" [Title/Abstract] OR "feeling*" [Title/Abstract] OR "Attitude" [MeSH Terms] OR "attitude*" [Title/Abstract] OR "opinion*" [Title/Abstract] OR "view*" [Title/Abstract] OR "perspective" [Title/Abstract] OR "need*" [Title/Abstract] OR "demand" [Title/Abstract] OR "requirement*" [Title/Abstract] OR "expectation*" [Title/Abstract]  #4 "Qualitative Research" [MeSH Terms] OR "qualitative*" [Title/Abstract] OR "qualitative study" [Title/Abstract] OR "qualitative interview" [Title/Abstract] OR "phenomenol*" [Title/Abstract] OR "phenomenological study" [Title/Abstract] OR "phenomenological research" [Title/Abstract] OR "hermeneutic*" [Title/Abstract] OR "Grounded Theory" [MeSH Terms] OR "grounded theory" [Title/Abstract] OR "ethnographic research" [Title/Abstract] OR "ethnological research" [Title/Abstract] OR "ethnonurs*" [Title/Abstract] OR "ethnomethodolog*" [Title/Abstract] OR "ethnograph*" [Title/Abstract] OR "Narration" [MeSH Terms] OR "narrati*" [Title/Abstract] OR "narrative interview" [Title/Abstract] OR "narrative study" [Title/Abstract] OR "action research" [Title/Abstract] OR "fieldwork" [Title/Abstract] OR "field work" [Title/Abstract] OR "field stud*" [Title/Abstract] OR " Interview "[Publication Type] OR "Interviews as Topic" [MeSH Terms] OR "interview*" [Title/Abstract] OR "Focus Groups" [MeSH Terms] OR "Focus Groups" [Title/Abstract] OR "content analysis" [Title/Abstract] OR "thematic analysis" [Title/Abstract] OR "mixed method*" [Title/Abstract] OR "mixed model*" [Title/Abstract] OR "mixed design*" [Title/Abstract] OR "multiple method*" [Title/Abstract]  #5 #1 AND #2 AND #3 AND #4 |
| **Embase** | #1 'cognitive defect'/exp/mj OR 'cognitive defect' OR 'mild cognitive impairment'/exp/mj OR 'mild cognitive impairment' OR 'cognitive decline'/exp/mj OR 'cognitive decline' OR 'mental deterioration'/exp/mj OR 'mental deterioration' OR 'disorders of higher cerebral function'/exp/mj OR 'disorders of higher cerebral function' OR 'cognitive defect':ab,ti OR 'cognitive dysfunction':ab,ti OR 'mild cognitive dysfunction':ab,ti OR 'mcd':ab,ti OR 'cognitive impairment':ab,ti OR 'mild cognitive impairment':ab,ti OR 'mci':ab,ti OR 'cognitive disorder':ab,ti OR 'cognition disorders':ab,ti OR 'mild cognitive disorder':ab,ti OR 'cognitive decline':ab,ti OR 'mild cognitive decline':ab,ti OR 'mental deterioration':ab,ti OR 'disorders of higher cerebral function':ab,ti OR 'neurocognitive disorder':ab,ti OR 'mental disorders':ab,ti OR 'neurocognitive disorders':ab,ti  #2 'diabetes mellitus'/exp/mj OR 'insulin dependent diabetes mellitus'/exp/mj OR 'non insulin dependent diabetes mellitus'/exp/mj OR 'diabetes mellitus':ab,ti OR 'dm':ab,ti OR 'diabet*':ab,ti OR 'diabetes mellitus, type 1':ab,ti OR 'diabetes mellitus,insulin-dependent':ab,ti OR 'iddm':ab,ti OR 'type 1 diabetes':ab,ti OR 't1dm':ab,ti OR 'diabetes mellitus, type 2':ab,ti OR 'diabetes mellitus,noninsulin-dependent':ab,ti OR 'type 2 diabetes':ab,ti OR 't2dm':ab,ti OR 'niddm':ab,ti  #3 'perception'/exp/mj OR 'attitude'/exp/mj OR 'experience'/exp/mj OR 'demand'/exp/mj OR 'expectation'/exp/mj OR 'perception*':ab,ti OR 'attitude*':ab,ti OR 'experien*':ab,ti OR 'demand':ab,ti OR 'expectation*':ab,ti OR 'feeling*':ab,ti OR 'opinion*':ab,ti OR 'view*':ab,ti OR 'perspective':ab,ti OR 'need*':ab,ti OR 'requirement*':ab,ti  #4 'qualitative research'/exp/mj OR 'grounded theory'/exp/mj OR 'interview'/exp/mj OR 'qualitative interview'/exp/mj OR 'phenomenology'/exp/mj OR 'ethnographic research'/exp/mj OR 'action research'/exp/mj OR 'field work'/exp/mj OR 'field study'/exp/mj OR 'content analysis'/exp/mj OR 'thematic analysis'/exp/mj OR 'mixed method'/exp/mj OR 'mixed model'/exp/mj OR 'qualitative*':ab,ti OR 'qualitative study':ab,ti OR 'qualitative research':ab,ti OR 'qualitative interview':ab,ti OR 'phenomenol*':ab,ti OR 'phenomenological study':ab,ti OR 'phenomenological research':ab,ti OR 'hermeneutic*':ab,ti OR 'grounded theory':ab,ti OR 'ethnographic research':ab,ti OR 'ethnological research':ab,ti OR 'ethnonurs*':ab,ti OR 'ethnomethodolog*':ab,ti OR 'ethnograph*':ab,ti OR 'narration':ab,ti OR 'narrati*':ab,ti OR 'narrative interview':ab,ti OR 'narrative study':ab,ti OR 'action research':ab,ti OR 'fieldwork':ab,ti OR 'field work':ab,ti OR 'field stud*':ab,ti OR 'interviews as topic':ab,ti OR 'interview*':ab,ti OR 'focus groups':ab,ti OR 'content analysis':ab,ti OR 'thematic analysis':ab,ti OR 'mixed method*':ab,ti OR 'mixed model*':ab,ti OR 'mixed design*':ab,ti OR 'multiple method*':ab,ti  #5 #1 AND #2 AND #3 AND #4 |
| **Web of Science** | TS=(("Cognitive Dysfunction" OR "mild cognitive dysfunction" OR "cognitive impairment" OR "mild cognitive impairment" OR "MCI" OR "cognitive disorder" OR "Cognition Disorders" OR "mild cognitive disorder" OR "cognitive decline" OR "mild cognitive decline" OR "MCD" OR "cognitive defect" OR "Neurocognitive Disorders" OR "neurocognitive disorder" OR "mental deterioration" OR "Mental Disorders" OR "neurocognitive disorders") AND ("Diabetes Mellitus" OR "Diabetes Mellitus, Type 1" OR "Diabetes Mellitus, Type 2" OR "DM" OR "diabet*" OR "Diabetes Mellitus,Insulin-Dependent" OR "IDDM" OR "type 1 diabetes" OR "T1DM" OR "Diabetes Mellitus,Noninsulin-Dependent" OR "type 2 diabetes" OR "T2DM" OR "NIDDM") AND ("experien*" OR "Perception" OR "perception*" OR "feeling*" OR "Attitude" OR "attitude*" OR "opinion*" OR "view*" OR "perspective" OR "need*" OR "demand" OR "requirement*" OR "expectation*") AND ("Qualitative Research" OR "qualitative*" OR "qualitative study" OR "qualitative interview" OR "phenomenol*" OR "phenomenological study" OR "phenomenological research" OR "hermeneutic*" OR "Grounded Theory" OR "ethnographic research" OR "ethnological research" OR "ethnonurs*" OR "ethnomethodolog*" OR "ethnograph*" OR "Narration" OR "narrati*" OR "narrative interview" OR "narrative study" OR "action research" OR "fieldwork" OR "field work" OR "field stud*" OR "Interviews as Topic" OR "interview*" OR "Focus Groups" OR "content analysis" OR "thematic analysis" OR "mixed method*" OR "mixed model*" OR "mixed design*" OR "multiple method*")) |
| **The Cochrane Library** | #1 MeSH descriptor: [Cognitive Dysfunction] explode all trees  #2 MeSH descriptor: [Neurocognitive Disorders] explode all trees  #3 MeSH descriptor: [Cognition Disorders] explode all trees  #4 MeSH descriptor: [Mental Disorders] explode all trees  #5 (cognitive dysfunction):ti,ab,kw OR (mild cognitive dysfunction):ti,ab,kw OR (cognitive impairment):ti,ab,kw OR (mild cognitive impairment):ti,ab,kw OR (MCI):ti,ab,kw  #6 (cognitive disorder):ti,ab,kw OR (cognition disorders):ti,ab,kw OR (mild cognitive disorder):ti,ab,kw OR (cognitive decline):ti,ab,kw OR (mild cognitive decline):ti,ab,kw  #7 (MCD):ti,ab,kw OR (cognitive defect):ti,ab,kw OR (neurocognitive disorders):ti,ab,kw OR (neurocognitive disorder):ti,ab,kw OR (mental disorders):ti,ab,kw  #8 (mental deterioration):ti,ab,kw  #9 #1 OR #2 OR #3 OR #4 OR #5 OR #6 OR #7 OR #8  #10 MeSH descriptor: [Diabetes Mellitus] explode all trees  #11 MeSH descriptor: [Diabetes Mellitus, Type 1] explode all trees  #12 MeSH descriptor: [Diabetes Mellitus, Type 2] explode all trees  #13 (diabetes mellitus):ti,ab,kw OR (DM):ti,ab,kw OR (diabet*):ti,ab,kw OR (Diabetes Mellitus, Type 1):ti,ab,kw OR (Diabetes Mellitus,Insulin-Dependent):ti,ab,kw  #14 (IDDM):ti,ab,kw OR (type 1 diabetes):ti,ab,kw OR (T1DM):ti,ab,kw OR (Diabetes Mellitus, Type 2):ti,ab,kw OR (Diabetes Mellitus,Noninsulin-Dependent):ti,ab,kw  #15 (type 2 diabetes):ti,ab,kw OR (T2DM):ti,ab,kw OR (NIDDM):ti,ab,kw  #16 #10 OR #11 OR #12 OR #13 OR #14 OR #15  #17 MeSH descriptor: [Perception] explode all trees  #18 MeSH descriptor: [Perception] explode all trees  #19 (perception*):ti,ab,kw OR (experien*):ti,ab,kw OR (feeling*):ti,ab,kw OR (attitude*):ti,ab,kw OR (opinion*):ti,ab,kw  #20 (view*):ti,ab,kw OR (perspective):ti,ab,kw OR (need*):ti,ab,kw OR (demand):ti,ab,kw OR (requirement*):ti,ab,kw  #21 (expectation*):ti,ab,kw  #22 #17 OR #18 OR #19 OR #20 OR #21  #23 MeSH descriptor: [Qualitative Research] explode all trees  #24 MeSH descriptor: [Hermeneutics] explode all trees  #25 MeSH descriptor: [Grounded Theory] explode all trees  #26 MeSH descriptor: [Narration] explode all trees  #27 MeSH descriptor: [Interviews as Topic] explode all trees  #28 MeSH descriptor: [Focus Groups] explode all trees  #29 (qualitative*):ti,ab,kw OR (qualitative research):ti,ab,kw OR (qualitative study):ti,ab,kw OR (qualitative interview):ti,ab,kw OR (phenomenol*):ti,ab,kw  #30 (phenomenological study):ti,ab,kw OR (phenomenological research):ti,ab,kw OR (hermeneutic*):ti,ab,kw OR (grounded theory):ti,ab,kw OR (ethnographic research):ti,ab,kw  #31 (ethnological research):ti,ab,kw OR (ethnonurs*):ti,ab,kw OR (ethnomethodolog*):ti,ab,kw OR (ethnograph*):ti,ab,kw OR (narrati*):ti,ab,kw  #32 (narrative interview):ti,ab,kw OR (narrative study):ti,ab,kw OR (action research):ti,ab,kw OR (fieldwork):ti,ab,kw OR (field work):ti,ab,kw  #33 (field stud*):ti,ab,kw OR (interview*):ti,ab,kw OR (Interviews as Topic):ti,ab,kw OR (Focus Groups):ti,ab,kw OR (content analysis):ti,ab,kw  #34 (thematic analysis):ti,ab,kw OR (mixed method*):ti,ab,kw OR (mixed model*):ti,ab,kw OR (mixed design*):ti,ab,kw OR (multiple method*):ti,ab,kw  #35 #23 OR #24 OR #25 OR #26 OR #27 OR #28 OR #29 OR #30 OR #31 OR #32 OR #33 OR #34  #36 #9 AND #16 #22 AND #35 |
| **CINAHL** | #1 MH diabetes meilltus OR MH Diabetes Mellitus, Type 1 OR diabetes mellitus type 2 OR AB diabetes mellitus OR AB diabet? OR AB dm OR AB type 1 diabetes OR AB type 2 diabetes OR AB Diabetes Mellitus,Noninsulin-Dependent OR AB Diabetes Mellitus,Insulin-Dependent OR AB t1dm OR AB t2dm  #2 MH cognitive dysfunction OR AB cognitive dysfunction OR AB mild cognitive dysfunction OR AB cognitive impairment OR AB mild cognitive impairment OR AB MCI OR AB cognitive disorders OR AB cognition disorders OR AB mild cognitive disorder OR AB cognitive decline OR AB mild cognitive decline OR AB MCD  #3 AB cognitive defect OR MH neurocognitive disorder OR AB neurocognitive disorder OR AB mental deterioration OR AB Mental Disorders OR AB neurocognitive disorders  #4 #2 OR #3  #5 AB experien？ OR MH Perception OR AB perception？ OR AB feeling OR MH Attitude OR AB attitude？ OR AB opinion？ OR AB view？ OR AB perspective OR AB need？ OR AB demand OR AB requirement？  #6 (AB expectation？) OR (#5)  #7 MH qualitative research OR AB qualitative? OR AB qualitative study OR AB qualitative interview OR AB phenomenol？ OR AB phenomenological study OR AB phenomenological research OR AB hermeneutic？ OR MH Grounded Theory OR AB grounded theory OR AB ethnographic research OR AB ethnological research  #8 AB ethnonurs？ OR AB ethnomethodolog？ OR AB ethnograph？ OR MH Narration OR AB phenomenol？ OR AB narrati？ OR AB narrative interview OR AB narrative study OR AB action research OR AB fieldwork OR AB field work OR AB field stud？  #9 AB Interview OR MH Interviews as Topic OR AB interview？ OR MH Focus Groups OR AB Focus Groups OR AB content analysis OR AB thematic analysis OR AB mixed method？ OR AB mixed model？ OR AB mixed design OR AB multiple method？  #10 #7 OR #8 OR #9  #11 #1 AND #4 AND #6 AND #10 |
| **PsycINFO** | #1 ("cognitive dysfunction" or "mild cognitive dysfunction" or "cognitive impairment" or "mild cognitive impairment" or "MCI" or "cognitive disorder" or "cognition disorders" or "mild cognitive disorder" or "cognitive decline" or "mild cognitive decline" or "MCD" or "cognitive defect" or "neurocognitive disorders" or "neurocognitive disorder" or "mental deterioration" or "mental disorders").ab,ti.  #2 ("diabetes mellitus" or "Diabetes Mellitus, Type 1" or "Diabetes Mellitus, Type 2" or "diabetes mellitus" or "DM" or "diabet*" or "Diabetes Mellitus,Insulin-Dependent" or "IDDM" or "type 1 diabetes" or "T1DM" or "Diabetes Mellitus,Noninsulin-Dependent" or "type 2 diabetes" or "T2DM" or "NIDDM" or "mental disorders").ab,ti.  #3 ("experien*" or "perception*" or "feeling*" or "attitude*" or "opinion*" or "view*" or "perspective" or "need*" or "demand" or "requirement*" or "expectation*").ab,ti.  #4 ("Qualitative Research" or "qualitative*" or "qualitative study" or "qualitative interview" or "phenomenol*" or "phenomenological study" or "phenomenological research" or "hermeneutic*" or "grounded theory" or "ethnographic research" or "ethnological research" or "ethnonurs*" or "ethnomethodolog*" or "ethnograph*" or "Narration" or "narrati*" or "narrative interview" or "narrative study" or "action research" or "fieldwork" or "field work" or "field stud*" or "Interviews as Topic" or "interview*" or "Focus Groups" or "content analysis" or "thematic analysis" or "mixed method*" or "mixed model*" or "mixed design*" or "multiple method*" or "Qualitative Methods").ab,ti.  #5 #1 and #2 and #3 and #4 |
| **WanFang** | 主题:((“糖尿病” or “1型糖尿病” or “2型糖尿病” or “胰岛素依赖型糖尿病” or “非胰岛素依赖型糖尿病”) and (“认知功能障碍” or “轻度认知功能障碍" or “认知障碍" or “轻度认知障碍" or “认知功能损害” or “轻度认知功能损害" or “认知功能减退” or “轻度认知功能减退" or “认知功能受损” or “认知损伤” or “认知功能下降” or “神经认知障碍” or “轻度神经认知障碍") and (“体验" or “经历” or “认知” or “感受” or “态度” or “需求”) and (“质性研究” or “定性研究” or “现象学" or “扎根理论” or “民族志” or “人种学研究” or “叙事研究” or “行动研究” or “田野研究” or “访谈” or “焦点群体访谈” or “主题分析” or “内容分析” or “混合研究”)) |
| **CNKI** | (TKA='认知功能障碍' OR TKA='轻度认知功能障碍' OR TKA='认知障碍' OR TKA='轻度认知障碍' OR TKA='认知功能损害' OR TKA='轻度认知功能损害' OR TKA='认知功能减退' OR TKA='轻度认知功能减退' OR TKA='认知功能受损' OR TKA='认知损伤' OR TKA='认知功能下降' OR TKA='神经认知障碍' OR TKA='轻度神经认知障碍') AND (TKA='糖尿病' OR TKA='1型糖尿病' OR TKA='2型糖尿病' OR TKA='胰岛素依赖型糖尿病' OR TKA='非胰岛素依赖型糖尿病') AND (TKA='体验' OR TKA='经历' OR TKA='认知' OR TKA='感受' OR TKA='态度' OR TKA='需求') AND (TKA='质性研究' OR TKA='定性研究' OR TKA='现象学' OR TKA='扎根理论' OR TKA='民族志' OR TKA='人种学研究' OR TKA='叙事研究' OR TKA='行动研究' OR TKA='田野研究' OR TKA='访谈' OR TKA='焦点群体访谈' OR TKA='主题分析' OR TKA='内容分析' OR TKA='混合研究') |
| **VIP** | #1糖尿病+1型糖尿病+2型糖尿病+胰岛素依赖型糖尿病+非胰岛素依赖型糖尿病  #2认知功能障碍+轻度认知功能障碍+认知障碍+轻度认知障碍+认知功能损害+轻度认知功能损害+认知功能减退+轻度认知功能减退+认知功能受损+认知损伤+认知功能下降+神经认知障碍+轻度神经认知障碍  #3体验+经历+认知+感受+态度+需求  #4质性研究+定性研究+现象学+扎根理论+民族志+人种学研究+叙事研究+行动研究+田野研究+访谈+焦点群体访谈+主题分析+内容分析+混合研究 |

# Supplementary table2. Qualitative synthesis Themes, sub-themes, and inclusion in papers

| **Analytical themes** | **Sub-themes** | **Descriptive themes** | **Illustrative quotations from participants** | **Studies that included** |
| --- | --- | --- | --- | --- |
| **1 Self-perception of cognitive impairment** | **1.1 Underestimate of cognitive decline associated with diabetes** | 1.1.1 Unclear impact of diabetes on cognitive function | “I never knew that diabetes was related to memory . . . that’s surprising.”  Another “never knew” that diabetes was “related to memory or cognitive stuff.” | Cuevas（2017） |
|  |  |  | “I never thought diabetes could have anything to do with memory loss, it's so strange.” | Hu（2019） |
|  |  | 1.1.2 Attributing cognitive problems to ageing | “Most of the people I see who are really old…didn’t start losing their brain until they were older. I think that’s…what just happens. We’re healthy until we’re not.”  “Sometimes I just have problems. It’s not the worst. I feel like this is worse now that I’m older.”  “Everyone my age has some kind of problem.” | Cuevas（2021） |
|  |  |  | Most attributed their perceived problems to “normal aging.” | Cuevas（2017） |
|  |  |  | “This was just a normal part of getting old.” | Cuevas（2018） |
|  |  |  | None thought these changes were related to diabetes, although the respondents, who were on the younger side, agreed that these cognitive changes were a normal part of the ageing process. | Hu（2019） |
|  |  | 1.1.3 Not aware that cognitive problems have arisen | “I might start to feel my brain’s not quite working properly… It’s the mental side of things when I’m slowing down and I notice that things aren’t quite right”.  “It’s just like a feeling of weakness… I tend to be a bit silly”.  None of the complaints were felt to be “hazardous to health.” Most were “common, everyday stuff,” related to forgetting names, misplacing items, or difficulty retaining new information. | Cuevas（2021） |
|  |  | 1.1.4 Lack of knowledge and unavailability of doctors | “No one talks to me about my brain.” | Cuevas（2021） |
|  |  |  | “If the doctor hadn't talked to me, I would never have known in my life that my poor memory now could be caused by diabetes.”  “After my first episode of hypoglycaemia I went online to find out that low blood sugar can cause a lack of energy supply to brain cells, but when my blood sugar came up my symptoms got better, and when I went to the doctor later, he didn't specifically tell me about diabetes causing cognitive dysfunction, not knowing that hypoglycaemia can affect memory if it happens more often.” | Hu（2019） |
|  |  |  | “I feel like I was not sure what kinds of questions to ask in the class. No one has ever talked to me before about how diabetes could change how well I think.”  “I had no idea I could do things to help my brain. Not all the cognitive strategies are things I’ve been able to do, but I feel like I’m making small changes and they are super useful.” | Cuevas（2018） |
|  |  | 1.1.5 Initial understanding of how hypoglycaemia affects memory | “But as the glucose changes there is a misfiring of neurons in the brain because there’s not enough sugar bouncing around up there. So somebody could be talking in the background and you may have heard it, but you won’t remember.” | Cuevas（2017） |
|  | **1.2 Suffering from cognitive symptoms** | 1.2.1 Deterioration of memory capacity | “I think short-term memory to be more specific. It’s the fact that I will try to do something, but I will say no I’ll do it in a minute and then I’ll forget and I’ll say wait, what’s happening. So I would classify it as short-term memory. Sometimes and this is something. I’m trying to think of a specific fact, for example, the name of an actor. Eventually I’ll get it, but it’s seems like it’s harder now that, well since I was diagnosed. It’s frustrating because it’s not at my fingertips.”  “Well, I can remember when I used to drive for [bus company] one time I couldn’t even remember where I was at. I forgot. Wrong turns and stuff like that.”  “I think I can’t remember certain things that used to be easier. Especially before I had diabetes.” | Cuevas（2017） |
|  |  |  | “I forget too easily.” | Cuevas（2018） |
|  |  |  | “I stand in front of the cupboard and think “why am I here?” | Speight（2014） |
|  |  |  | “I think the short-term memory loss is the most prominent, there are things that I have to do right away that I turn around and forget what I was just going to do, these seem like things that should only happen to my mum and now they happen to me all the time, but I'm only a few years old.”  “Before I got sick I had a pretty good memory, now I often have some celebrity names that take me ages to remember, they used to be quite easy for me, so now I get a bit frustrated sometimes because my mind doesn't seem to be as controlled as it used to be.”  “I used to never forget a road I drove on once, but lately I've been driving the wrong way on some roads I don't drive very often, so I'm using navigation more and more when I go out.”  “I'm finding that I'm leaving my keys on the front door more and more.” | Hu（2019） |
|  |  | 1.2.2 Lack of attention | “Feeling of anxiety… light palpitation… concentration wanders…”  “Lack concentration, grate my teeth”.  “It’s just a fuzzy head type of thing. Lack of interest in anything but just total lack of concentration”. | Speight（2014） |
|  |  | 1.2.3 Sickness shame | Others were concerned with slow speed of processing and mentioned they would “stay quiet because people will think I’m stupid if I can’t come up with an answer quickly.” | Cuevas（2018） |
|  |  | 1.2.4 Difficult to think | “My brain would turn to jelly.” | Speight（2014） |
|  |  |  | “It's not very nice when you can't think because your brain fog.” | Chepulis（2021） |
|  |  | 1.2.5 slow response | “They were not as sharp as they used to be.” | Wilson（2012） |
|  | **1.3 Impaired diabetes self-management** | 1.3.1 difficult to apply new knowledge | Cognitive impairment leads to difficulties in applying new knowledge to the daily routine.  Impairment of cognitive skills and competencies represents a barrier in the adequate application and implementation of new knowledge to the lives of those concerned. | Hasseler（2011） |
|  |  | 1.3.2 Cognitive problems affect the application of drugs | “I think I have problems remembering to take my medications, but it does not benefit me to mark on calendars or anything like that. I even have a pill that I have to take an hour after I eat and I’ll remember it past the hour and it’s too late.” | Cuevas（2017） |
|  |  |  | “I often forget to take my medication on time now, even if I write it down on my calendar I still forget, I remember one time I delayed taking my medication by over an hour, it seemed a bit too late and my blood sugar was bad that day.”  “I used to forget my pre-meal insulin and have to make it up after the meal, now sometimes after the meal I can't remember if I just took it or not and I feel a bit scared just thinking about it.” | Hu（2019） |
|  |  |  | “Cause man, half the time I don't remember to take my pills.”  “Sometimes I have forgotten, like today. Normally I am in a habit of putting the [insulin] pen on the table which I forgot to do this morning.” | Chepulis（2021） |
|  |  |  | “I often find that I forget things like testing my sugars or taking my insulin on time. It has been a problem in the past when I’ve fallen asleep without any insulin and made myself ill. I had to go into hospital one time because I started to be sick. They said my sugar levels were very high”. | Wilson（2012） |
|  |  | 1.3.3 Cognitive problems affect diet | “Planning what to eat is hard . . . I always have to think ahead about what am I gonna eat. And am I going to eat this or that or when. And a lot of times I don’t think ahead.” | Cuevas（2017） |
|  |  |  | “I often think about what I want to eat the next day, but now I often get confused about whether I should eat these dishes for lunch or in the evening, I had a plan, but now I think about it for nothing, forget it, I'll just eat what I have. ” | Hu（2019） |
|  |  |  | “I can’t find my way from A to B, so when people say ‘go into the kitchen and get something to eat,’ that’s fine but I don’t know where my kitchen is.” | Speight（2014） |
|  |  | 1.3.4 Cognitive issues affect disease management | “I have problems thinking about what to do next because there are so many things in diabetes to take care of.” | Cuevas（2021） |
|  |  |  | “I was diagnosed with early dementia a few years ago and find that I don’t always remember to do what I should to look after myself properly. I didn’t take my diabetic tablets one time and I felt unwell. I often find it difficult to manage with a poor memory and diabetes because I live on my own.” | Wilson（2012） |
|  |  | 1.3.5 Cognitive issues affect caregiving families | “I have to make sure I’m on top of everything. I have a family to take care of.” | Cuevas（2021） |
|  |  | 1.3.6 Cognitive problems affect work | “My job is to work as hard as I can so I can support my family. If I can’t think straight, I can’t work.” | Cuevas（2021） |
|  |  | 1.3.7 Cognitive problems affect the maintenance of social relationships | “Hispanic people. You know. We like to have family parties and eat. If someone doesn’t want to come over for a holiday. Something is wrong with their mind, no?” | Cuevas（2021） |
|  | **1.4 Coping in multiple methods** | 1.4.1 Adding to knowledge by reading | “I need to read up and some doctors don’t say anything. I need to learn. And you know with diabetes there are some things you can get like neuropathy and so any type of change in your memory it’s probably related to the diabetes.” | Cuevas（2017） |
|  |  | 1.4.2 Use the internet to access relevant information | “I use the Internet more to find out information on how to keep my brain healthy.” | Cuevas（2021） |
|  |  | 1.4.3 Use of cognitive strategies to maintain cognitive function | “When I was still working and I was at the register at the pharmacy. And I would look around the store and I would get a word and see how many words I could make out of that one. I would write it on a piece of paper.” | Cuevas（2017） |
|  |  |  | “It takes a lot. If I think of everything I have to do all at once, I know I forget things. So I now I make a list of groceries and then I quit checking my phone while I’m at [grocery store].” | Cuevas（2018） |
|  |  |  | “I use a timer on my phone so I know I only have to pay attention for 25 min at work and then I can take a quick break.”  “I keep lists. Because if I don’t write it down, I forget it.”  “these are just things I do to help me get work done.” | Cuevas（2021） |
|  |  | 1.4.4 Keeping the hobby alive | It would be very easy for the person who has a memory problem not to remember what those terms [for sewing] are. But with practicing it brings in not only the kinetic of doing something with your hands, but then the mental piece too. It needs to be repetitive stuff. So if you make a potholder you’re going to learn to make three. | Cuevas（2017） |
|  |  | 1.4.5 Diet control | “I need to eat less junk food to get rid of the brain fog.”  “I need a diet for diabetes and maybe it will help my brain. I think I could learn that in a class.” | Cuevas（2017） |
|  |  | 1.4.6 Activating the brain | “Not letting my brain get bored, being able to learn new things.” | Cuevas（2021） |
|  |  | 1.4.7 Keep working | “As long as I can keep working and not make mistakes…my brain will be healthy.” | Cuevas（2021） |
|  |  | 1.4.8 Sports activities | Physical activity “kept blood moving to the brain” . | Cuevas（2021） |
|  |  | 1.4.9 Electronic games | The topic of games was brought up by participants as something to “help my mind,” | Cuevas（2021） |
|  |  | 1.4.10 Maintaining socialization | “I need to work hard to maintain the connections I have. If I don’t, I’ll forget how to act, how to do things.” | Cuevas（2021） |
| **2 PWDs reported benefits of cognitive interventions** | **2.1 Attitude to cognitive interventions** | 2.1.1 Attitudes towards cognitive interventions | “I wanted my brain to change in a better way. And I think this may be one way to do it. It takes a lot of work, but I think with exercise, diet, paying attention to my sugar and practicing strategies, I can at least help my brain not get worse.” | Cuevas（2017） |
|  |  |  | “It’s hard to know what I need to do when the information and advertising is misleading. And I get that there’s still more research that’s needed. There’s so much out that trying to sell me things. It helped to have someone in health care clear it up for us.”  “I’m glad to do something like this. I see ads on TV all the time for vitamins for my brain, but I don’t know what works. This helps a lot and makes me think better. I like sharing with other people what I work and then I can go to my doctor too and talk about what I’ve been doing.” | Cuevas（2018） |
|  |  |  | All participants said that they would be willing to commit to an intervention of 4 to 6 weeks in length; they thought they would be able to adhere to a 45-minute, 3 times per week online game schedule. | Cuevas（2017） |
|  |  | 2.1.2 Other facilitating factors | Ease of use of online games, accessibility, and convenient scheduling of group sessions were mentioned as important aspects of an intervention. | Cuevas（2017） |
|  |  | 2.1.3 Motivation to use cognitive strategies | “When I see my physician, she tells me to keep trying to be healthy and to keep us the good work.”  “I realized that if I don’t want to get worse diabetes then I have to take control of my sugars. But I can’t always remember what I need to do next. These classes helped me figure out ways to think about what I’m doing and then make a list or set my phone alarm. It’s motivation to be think in better ways so my diabetes can be better. . .and my diabetes nurse can help me.” | Cuevas（2018） |
|  |  | 2.1.4 Lack of a plan or difficulty in implementing a plan | “I have every intention to exercise. I make time in my schedule, but something always comes up.”  “I need more than just writing down one goal. Sometimes I never look at it again.”"  “I have problems planning, I can’t think that far ahead.” | Cuevas（2018） |
|  |  | 2.1.5 Difficult to change habits | “One more thing is being asked of me and I do not know if I can do any more.” | Cuevas（2018） |
|  |  | 2.1.6 Other Barriers | Potential barriers to participation included travel to group sessions and timing of the classes (ie, interference with work hours. | Cuevas（2017） |
|  |  |  | “I think I would come to a class like this if it was available, I'm just worried that I won't have time when it comes to classes.” | Hu（2019） |
|  | **2.2 Preference for cognitive intervention** | 2.2.1 Better cognitive strategies needed to help with disease management | “I need to plan better. I can’t remember as well.” | Cuevas（2021） |
|  |  |  | “I think the computer training was fun, but I like working on the cognitive strategies better. I spent more time on making lists and stopping to think things through than on video games.” | Cuevas（2018） |
|  |  | 2.2.2 Thirst for knowledge on diabetes-related cognitive impairment | “It’s important to know so you can be prepared and you cannot think you’re going crazy.” | Cuevas（2017） |
|  |  |  | “I would like to learn something about the diabetic diet that will help me improve my memory, preferably where I can listen to a class.”  “I want to know why diabetes causes memory loss, how do I know my cognitive function is getting worse and what can I do to keep my brain healthy?”  “I don't want to become demented. Is there any medicine I can take? But it seems a bit early to be taking medication.” | Hu（2019） |
|  |  | 2.2.3 Desire for cognitive interventions that can improve lives | They recommended a continued focus on teaching cognitive strategies “to make life easier.”  Specific areas of interest for intervention class content included the following: understanding how cognitive function relates to diabetes, dealing with cognitive barriers to diabetes self-management, discussing how stress associated with diabetes is related to cognitive function, and learning how to incorporate a “brain healthy” lifestyle, particularly related to diet, into daily activities. | Cuevas（2017） |
|  |  | 2.2.4 Looking for peers to share | All expected to “learn cognitive strategies from each other and share ideas” and preferred a group format.  “I wasn’t sure what would work and what wouldn’t. I didn’t anticipate that most people had tried different things, and I want to continue talking about this with others.” | Cuevas（2017） |
|  |  | 2.2.5 Desire for exercises to reduce anxiety and improve concentration | They recommended more emphasis on practices such as meditation and deep breathing to help improve focus and decrease the anxiety they associated with diabetes management.  One said the meditation practice taught in a session helped to “decrease my anxiety and let me think more clearly about what I needed to do that day. I think anxiety was a big piece of what was keeping me from working on my diabetes and I didn’t realize it before.” | Cuevas（2018） |
|  | **2.3 Benefiting from cognitive interventions** | 2.3.1 Be able to apply the content of the intervention | They would be able to learn strategies and put them into practice and improvement would take time that extended beyond taking part in the intervention. | Cuevas（2017） |
|  |  | 2.3.2 Proactively seek help to improve cognitive function | They now had questions for their health-care providers such as checking for vitamin B12 deficiency related to metformin use or tests that would be useful in investigating other causes of cognitive problems. | Cuevas（2018） |
|  |  | 2.3.3 Promotes thinking and the use of cognitive strategies | “These classes helped me figure out ways to think about what I’m doing and then make a list or set my phone alarm. It’s motivation to be think in better ways so my diabetes can be better.”  “This isn’t the solution to all my diabetes and thinking problems. But it’s a good foundation course that I think underlies a lot of other approaches to getting me to work more on thinking better and keeping my glucose controlled.” | Cuevas（2018） |
|  |  | 2.3.4 Fulfillment | Most had a sense of achievement  They felt the intervention helped increase their “mental abilities and flexibility with cognitive strategy plans.” | Cuevas（2018） |
|  |  | 2.3.5 Adherence to cognitive strategies to promote health after intervention | “The shorter goals were things that I knew I needed to do, but I had to keep in mind that they were for a bigger purpose. I need to keep doing these things and make them habits so I can be healthier.” | Cuevas（2018） |
